# A conversation with Vishva Dixit

**DOI:** 10.1172/JCI164341

**Published:** 2022-09-15

**Authors:** Ushma S. Neill

On any given day, over 50 billion of the cells in your body will die. The question of how we live while our cells are continuously dying has captivated Dr. Vishva Dixit. After a long career in academia, Dixit (Figure 1) rose through the ranks at Genentech and currently serves as the Vice President of Early Discovery Research. His scientific work has focused on the elucidation of the mechanisms of apoptosis, including the discovery of caspases. For the full interview, see https://www.jci.org/videos/cgms

*JCI*: Can you tell us a little bit about your parents and your childhood?

Dixit: I was born and brought up in Africa — in Kenya. My physician parents were of Indian origin; they were drafted into the British colonial service. This was in the 1940s, and they worked in the northern frontier district — the northern part of Kenya in the Sahara Desert that abuts Somalia. There were prisoner-of-war camps there, and it was largely inhabited by nomadic tribes. My father said it was characterized by the five s’s: sun, sand, scorpions, snakes, and shiftas (the local bandits). After the camps disbanded, they moved to the highlands in Kenya where the tea estates are. When I was born, Kenya was a colony: we had a very strict apartheid system. Blacks, Browns, and Whites were kept apart in schools and hospitals. It was an unstable time, but independence was achieved in 1963 and then things rapidly changed. There was integration of schools, and my parents were anxious to send me to what was previously the European school.

I was a fairly withdrawn, but happy child. I was withdrawn because I had congenital keratoconus, so I couldn’t see well. Later in life, I required corneal grafts to correct that. But I couldn’t participate in sports, so I found my joy in books — my outlet was reading about explorers, inventions, and the unknown.

*JCI*: Were you encouraged from a young age to go to medical school?

Dixit: There was a sense that doing medicine was a noble profession, but in all honesty, after independence in Africa, the way power had been consolidated by amplifying tribalism led to a lot of instability. There was a sense in the Indian community in East Africa, especially after Idi Amin came to power in Uganda, that maybe the younger generation needed a career that had a certain safety and acceptance. I was reluctant, but my brother Rajiv took me to the hospital and introduced me to medicine as a science. I gradually convinced myself that medicine was something I could do, but with a career in medical research.

*JCI*: Did you do research at University of Nairobi medical school?

Dixit: I was very fortunate to come under the influence of Professor Hettiaratchi, a professor of physiology. He emphasized constancy of the internal environment and Claude Bernard’s teachings about how systems maintain the status quo: one could take a liter of blood from somebody, yet their blood pressure would be unaltered because they’d compensate by employing vasoconstriction and an accelerated heart rate. What he really emphasized to me was that to learn complex systems, you have to break them down and make them simple.

We looked at electroencephalograms of children with subacute sclerosing panencephalitis who were either in a rural or urban environment to see if there was a correlation with where they lived. We also wrote a couple of letters to the journal *Medical Hypotheses* about nephrotic syndrome, vitamin C deficiency, and scurvy.

For a public health project, we were taken to a village with no running water or electricity. My research project was on the pathogen burden that people carry in these environments; with a blood smear and a stool specimen, you could look for malaria and intestinal parasites. The vast majority was highly anemic with a huge burden of pathogens that they just lived with.

*JCI*: How did you end up at Washington University for a pathology residency?

Dixit: I have to credit my brother. My brother somehow managed to get an internal medicine residency at Barnes Hospital at Wash U. He impressed them and encouraged me to join him. I was thinking, maybe I’d go to the UK to do a PhD. He mentioned the pathology program at Wash U was exceptionally enlightened, in that they allowed residents to do research.

My brother convinced the director of the program to give me a chance. For me, upon arrival, I was just struck by the affluence of the place: the hospital was carpeted, with individual patient rooms. Where I came from, there were days we had two patients to a bed and a patient in between. It was like I had travelled to another planet.

*JCI*: Tell us a little bit about your work with William Frazier on thrombosis.

Dixit: I was exceptionally naive, and even that may be an exaggeration. I was clueless. The department realized I would not be an attractive research assistant, as I hadn’t done any research. So they paid my stipend, so I’d be a free set of hands.

I interviewed with a lot of people, including a biochemist, Bill Frazier, who was working on how *Dictyostelium discoideum* (slime mold) adhere to each other. He posited that platelets aren’t that different and that they stick to each other. He asked me a series of questions on whether I’d ever used a PIPETMAN or a pH meter, to which I admitted only familiarity with litmus paper. Astonishingly, he consented to let me shadow him anyway. I became his apprentice, and he instilled a fearlessness in me. But we were flying by the seat of our pants in answering the question, What makes platelets adhere? We were particularly interested in this molecule thrombospondin, identified by Phil Majerus at Wash U years before, that maybe was an adhesion molecule, and our work showed it was.

*JCI*: You used that foundation to transition into a faculty position at the University of Michigan.

Dixit: I was recruited to the Department of Pathology by Peter Ward, who knew my heart wasn’t in clinical work. He pledged that if I could cover most of my salary from NIH grants, I could spend all my time in the lab. Gratifyingly, I got funded, and in short order, I was able to show that thrombospondin was made by mammalian cells — it was not only a platelet product, but also made by fibroblasts and endothelial cells where it played an adhesive role. There wasn’t just one thrombospondin; while I was in Ann Arbor, we cloned thrombospondin 2 and 3.

Ann Arbor at that time was quite exciting. Bill Kelley had recruited a great cadre of young researchers: Francis Collins, Craig Thompson, Jim Wilson, Gary and Betsy Nabel, Jeff Leiden, and John Lowe — the list goes on and on. They were doing incredible work, and they threw down the gauntlet. I looked at my own work, which was good, but I realized I wasn’t really breaking new territory.

*JCI*: How did you fixate on moving into the world of cell death and apoptosis?

Dixit: In the late 80s/early 90s, everybody was possessed by the cell cycle. I was aware of the work of Andrew Wyllie and John Kerr, who had described a form of cell death: apoptosis. I thought it interesting, but utterly unamenable to a biochemical analysis, because apoptosis happens in tissues, and you can’t isolate those cells; what was needed was a cell-culture system where you can induce apoptosis in a programmed manner.

Before going home, I would stop by the library where I read a paper by Yonehara in the *Journal of Experimental Medicine* and a paper by Peter Krammer in *Science*, describing antibodies to what we now know is the Fas receptor. These antibodies could induce cell death in culture. I wrote to Yonehara, who sent me the agonist antibody. The receptor meanwhile had been cloned by Shige Nagata and had homology to the TNF receptor — that had been characterized by David Goeddel. There was the realization that in certain cell systems, engagement of the TNF receptor by TNF could also lead to apoptosis. I wrote to Genentech, and I got recombinant TNF from them.

I had a really gifted MD-PhD student, Muneesh Tewari, and asked him to work on what was downstream of these “death receptors.” He found an MCF7 variant cell line that was sensitive to either the agonist antibody or TNF, and when he added these reagents, there would be this dance of death. But what was the signaling pathway? We decided to take an unbiased approach — what I referred to as the “Sigma catalog” approach. We were going to take kinase inhibitors/phosphatase inhibitors/you name it and throw it on the cells and look for positive selection: cells that would live in this ocean of death. If we found such a compound, maybe that would illuminate the downstream pathway. Two years went by, and we were most of the way through the Sigma catalog. Nothing.

Work going on in disparate fields culminated to give us a hint. There was work in the field of IL-1 processing by Nancy Thornberry and Roy Black. IL-1β and IL-18 are made in the pro form; to get the active cytokines, they need to be proteolytically processed. They were after the protease, because they thought if they could identify the protease and inhibit it, they could inhibit the emergence of active cytokines. They called this cysteine protease ICE, for interleukin-1 converting enzyme. At the same time, Bob Horowitz’s lab at MIT had cloned and identified the centrally important death gene in *C*. *elegans*, known as *Ced-3*. They had no idea what it did, and one day they ran it through the database and realized it had homology to ICE.

I asked Muneesh, “What if death receptors are engaging an ICE-like protease or ICE?” He recalled there was an inhibitor — a pox virus gene *CrmA* that was a serpin, but it inhibited ICE-like proteases. He encouraged me to write to David Pickup and Guy Salvesen at Duke University to get it. We got the expression construct and Muneesh expressed it that very day, and by the evening, cells that had formerly died in the presence of TNF or agonist antibodies were now happily growing when they expressed CrmA. We had completely nullified death. We knew then that downstream of the death receptors was an ICE or an ICE-like protease. Further work showed it to be an ICE-like protease that Muneesh called Yama (for the Hindu goddess of death), now known as caspase-3.

Once we found caspase-3, we had another major problem: how does the receptor, which the work of Goeddel and Nagata had shown possesses no enzymatic activity, activate Yama/caspase-3? I had another very gifted MD-PhD student join the lab, Arul Chinnaiyan, and a postdoc, Marta Muzio, who quickly unraveled this. Receptors were thought to work by either functioning as ion channels or by altering phosphorylation/dephosphorylation. They discovered, however, that death receptors were/did neither, but actually assembled a signaling complex that we dubbed the DISC (death-inducing signaling complex) that recruited an initiating ICE-like protease (now known as caspase-8). This was activated by a mechanism that Guy Salvesen and I called the “induced proximity model” that allowed the generation of an active protease as the second messenger.

*JCI*: Given these exciting advances, what made you want to leave for Genentech?

Dixit: I have a habit of throwing caution to the wind when it comes to science. My brother was a rheumatologist in the Bay Area, and I got a call from a headhunter who said there was a position (Director of Oncology) at Genentech. I thought it an opportunity to have dinner with my brother. When I came, however, I was very impressed by the CEO, Art Levinson, and the chief medical officer, Sue Hellman. They were really committed to cancer therapeutics and basic discovery.

Genentech is a very unique environment, and people often ask me, “What’s it like to be in industry?” I say, tongue in cheek, “I haven’t the foggiest because I work at Genentech. I don’t work in industry.” The ethos at Genentech is that if you do good basic research and you unveil fundamentally important components, then drug discovery will follow.

*JCI*: If you could not have been a physician or a scientist, what do you think might have kept you as motivated?

Dixit: I always wanted to be an explorer, so maybe an Egyptologist. Whatever it is that centers on discovering something new. New knowledge is what attracts me.

## Figures and Tables

**Figure 1:**
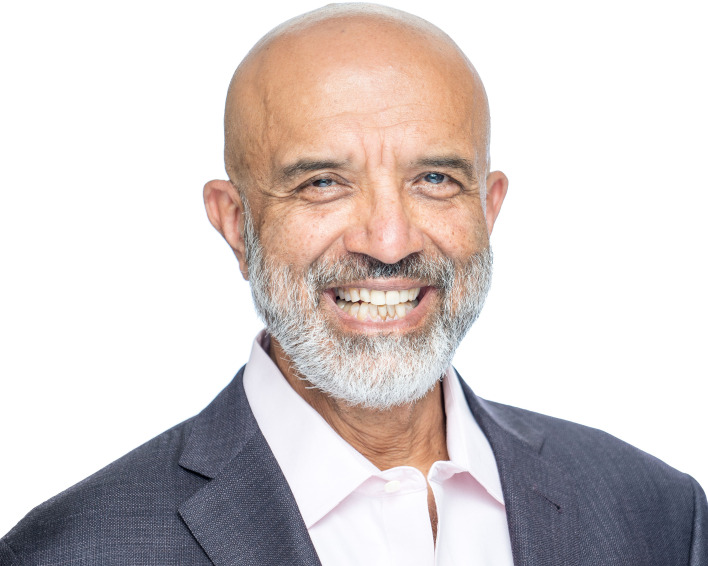
Vishva Dixit. Image credit: Genentech

## Version 1 09/15/2022

Electronic publication

